# Nitrogen addition shifts the microbial community in the rhizosphere of *Pinus tabuliformis* in Northwestern China

**DOI:** 10.1371/journal.pone.0172382

**Published:** 2017-02-24

**Authors:** Fenglian Lv, Sha Xue, Guoliang Wang, Chao Zhang

**Affiliations:** 1 State Key Laboratory of Soil Erosion and Dryland Farming on the Loess Plateau, College of Natural Resources and Environment, Northwest A & F University, Yangling, Shaanxi, China; 2 Institute of Soil and Water Conservation, Northwest A & F University, Yangling, Shaanxi, China; University of Oklahoma, UNITED STATES

## Abstract

Atmospheric nitrogen (N) deposition profoundly alters the soil microbial communities and will thus affect nutrient cycles. The effects of N availability on microbial community, however, are not clear. We used PLFA analysis to evaluate the effects of a gradient of N addition (0, 2.8, 5.6, 11.2, and 22.4 g N m^-2^ y^-1^) for three years on the rhizospheric microbial community of *Pinus tabuliformis* seedlings. The main factors influencing the community were quantified using structural equation modelling and redundancy analysis. At the microbial-community level, N addition increased the total phospholipid fatty acids content by increasing the dissolved organic carbon (DOC) and root biomass. Increases in soil microbial biomass carbon and N, however, was attributed to the increased DOC, N content and decreased pH. At the microbial-groups level, Fungal, arbuscular mycorrhizal fungal (AMF), gram-positive bacterial (GP) abundances and the GP:GN ratio first increased and then decreased with N addition. Nitrogen addition increased the abundances of bacteria, fungi, and actinomycetes mainly by increasing the DOC content and decreasing root biomass. Additionally, the decrease of pH and ammonium N caused by N addition increased the fungal abundances and reduced actinomycete abundances, respectively. Nitrogen addition shifted the rhizospheric microbial community mainly by altering the DOC content and root biomass. The current rate of N deposition (2.5 g N m^-2^ y^-1^) benefits plant growth and increases the abundances of fungi, arbuscular mycorrhizal fungi, GP, actinomycetes and the GP:GN ratio.

## Introduction

Recent anthropogenic activities (e.g. fossil-fuel combustion and application of artificial fertilisers) have dramatically increased the levels of available nitrogen (N) in soil ecosystems [1] and have substantially changed soil microbial communities in forest ecosystems. Changes in the composition of soil microbial communities, especially those of rhizospheric microbes, can affect plant-soil-microbe interactions and further alter terrestrial ecosystemic carbon (C) and N cycles and energy flow [2], with consequences for plant growth [3].

Nitrogen deposition is the main source of soil available N, and global inputs of N into terrestrial ecosystem have doubled over the last 100 years [4]. Nitrogen deposition increased from 1.3 g N m^-2^ y^-1^ in 1980 to 3.5 g N m^-2^ y^-1^ in 2012 in Northern China [5]. Low levels of N addition, particularly in N limited ecosystem, can generally mitigate N limitation [6] and increase plant biomass [7], but excessive N inputs can remarkably alter the soil physicochemical properties and influence the natural structure of soil by changing decomposition of soil organic matter, influencing formation of soil aggregate structure and compacting soil bulk density [8–10], which may negatively affect plant growth and be toxic to soil microbes [4]. Nitrogen deposition can alter the levels of soil available N and dissolved organic C (DOC) and decrease the soil pH of forest ecosystem [11], which will affect soil microbial communities and their biomass. For example, N deposition increased soil available-N content (e.g. NH_4_^+^-N and NO_3_^-^-N), which can cause changes in microbial communities [12], but reasons about the effect of increase in available N on different microbes (e.g. bacteria, fungi, and actinomycetes and their ratios) [12,13] have not been resolved.

Available-N enrichment generally alters fungal abundance, particularly arbuscular mycorrhizal fungi (AMF), and increases or has little effect on bacterial biomass [14]. The shift in soil available N has been correlated with DOC content [15]. Nitrogen addition has neutral or positive effects on soil DOC content, especially in rhizosphere [16] and may thus alter soil C and N dynamics, with further consequences for soil microbial communities. Change in DOC content is one of the main mechanisms for changing the composition of microbial (especially bacterial) communities [17,18]. Another study, however, reported that shifts in the composition of soil microbial communities from N addition were likely due to the decreased soil pH rather than to changes in the content of available N or DOC, and considered pH as a good indicator of the actual changes in soil microbes [19]. We hypothesised that the levels of ammonium N (NH_4_^+^-N), nitrate N (NO_3_^-^-N), and DOC could be the main factors changing the structure of soil microbial communities in N-limited regions and that soil pH may play a major role in oversaturated cases.

Nitrogen deposition, though, can affect soil microbial communities by altering plant growth, such as the allocation of aboveground and root biomass [20]. Studies in forests in Northern China and other ecosystems around the world have documented significant influences of plant productivity (aboveground and root biomass) on microbial community structure [16,21,22], especially increased in aboveground litter input as the main form of C resources for the growth of microbes. However, most experimental studies have focused on changes in plant growth or soil properties from N addition to explore the response of soil microbial communities, but the effects of the relationship between plants and soil properties under N deposition on the microbial communities are not clear. Also these effects need to be quantified to identify the main causes of changes in soil microbial communities.

Studying the effects of N addition, from limitations to oversaturation, is therefore important for identifying the mechanisms of the potential impacts on soil microbes and to predicting future implications of N deposition. We used *Pinus tabuliformis*, one of the most widespread tree species in Northwestern China and the main species for afforestation, as a test subject to study the effects of N addition gradient (from insufficient to an excess) on soil and plant characteristics and on different groups of microbes. We tested the hypothesis that N addition could affect the structure and composition of soil microbial communities in N-limited regions mainly by altering DOC content and root biomass.

## Materials and methods

### 2.1. Site description and experimental design

The experiment was conducted at the experimental field of the Institute of Soil and Water Conservation in Yangling, Shaanxi Province, China (108°40′27.95″E, 34°160′56.24″N). This area has a semi-arid climate with a mean annual temperature of 13.2°C and an average frost-free period of 225 d. The average annual precipitation is 674.3 mm, more than 60% of which falls between July and September.

We sowed *P*. *tabuliformis* seeds in a 5 × 10 m seedbed in March 2010. The N addition experiment began in March 2011 when the one-year-old seedlings were transplanted to PVC pots (35 cm deep × 40 cm in diameter) containing 18 kg of brown forest soil (Eutric Luvisd) from Yichuan, Shaanxi Province. The soil contained 13.5 g kg^-1^ organic matter, 1.54 g kg^-1^ total N, and 1.42 g kg^-1^ total P. The soil was passed through a 2-mm sieve and mixed with an N fertiliser (urea). The experiment had five N-addition treatments with 45 replicates each: 0 (CK), 0.057 (N_2.8_), 1.15 (N_5.6_), 2.3 (N_11.2_), and 4.6 (N_22.4_) g urea pot^-1^, equivalent to depositions of 0, 2.8, 5.6, 11.2, and 22.4 g N m^-2^ y^-1^, respectively. Each pot was packed to a bulk density of approximately 1.1 g cm^-3^. Corresponding dosages of urea were dissolved in 10 ml of distilled water and applied evenly to the pots during a rain at the end of March or in early April each year from 2012 to 2014.

We selected six *P*. *tabuliformis* seedlings with similar heights and ground diameters from each N treatment in mid-July 2014. The aboveground tissues and the roots of the seedlings were separated by clipping at the soil surface. The rhizospheric soil was collected using the shaking method [23] and placed in individual plastic bags, and the roots were stored in a cooler. All samples were transported to the laboratory, and residual roots, stones, and other debris were removed. The rhizospheric soil samples were passed through a 2-mm sieve. Subsamples were freeze-dried and stored at -86°C for determining the levels of phospholipid fatty acids (PLFAs). The remaining subsamples were immediately stored at 4°C for measuring soil physical and chemical properties, microbial biomass C (MBC), and microbial biomass N (MBN). We placed the soil samples under -86°C and 4°C for three and seven days before downstream analysis, respectively.

### 2.2. Plant biomass

The plant materials were oven-dried at 65°C for 48 h and weighed. We used the dry masses of the seedlings per pot averaged over six replicates for each treatment to estimate the production of aboveground biomass. Root samples were soaked in distilled water, cleaned of residual soil, and then oven-dried and weighed.

### 2.3. Soil properties

Soil moisture was determined as the loss of weight after drying the soil at 105°C for 24 h. Soil pH was measured in a 1:2.5 soil:water suspension using an automatic acid-base titrator. NO_3_^-^-N and NH_4_^+^-N were extracted with 50 ml of 2 M KCl, and the extract was analysed on an AutoAnalyzer III (Bran+Luebbe GmbH, Germany). DOC was extracted by adding 120 ml of 0.5 M K_2_SO_4_ to 40 g subsamples of homogenised soil and then with agitation on an orbital shaker at 120 rpm for 1 h. The filtrate was analysed using a TOC Analyser (Phoenix 8000, Tekmar Dohrmann, Mason, USA).

### 2.4. Microbial biomass and community composition

MBC and MBN were processed by fumigation/extraction using 15 g of soil and 0.5M K_2_SO_4_. MBC content was determined by TOC Analyzer, and MBN content was determined colorimetrically with a spectrophotometer (Hitachi, Tokyo, Japan, UV2300) at 220 and 275 nm. The MBC and MBN content were calculated using a K_EC_ factor of 0.45 and a K_EN_ factor of 0.54, respectively.

The PLFA analysis was based on the method of Bligh and Dyer [24] and modified by Bardgett et al. [25]. 3 g of freeze-dried soil was added to Teflon tubes containing a chloroform:methanol:citric acid buffer at a volumetric ratio of 1:2:0.8. Lipids and PLFAs were separated by silica-gel column chromatography, and the PLFAs were separated using gas chromatography/mass spectroscopy (N7890, Agilent, USA) after alkaline methylation analysis of all fatty-acid content, expressed as the mole fraction of fatty-acid methyl esters using the peak of an internal standard (19:0) as a reference. The abundances of the microbes and their biomasses were determined from the PLFA spectra. A mixture of bacterial fatty-acid methyl esters (FAMEs) ranging from C_11_ to C_20_ was used as a qualitative standard to identify the separated FAMEs (Supelco, UK). The branched, saturated PLFAs i15:0, a15:0, i16:0, i17:0, and a17:0 were chosen to represent gram-positive bacteria (GP), and 16:1w9, cy17:0, 18:1w9, cy19:0, and saturated fatty acids containing an -OH group were chosen to represent gram-negative bacteria (GN). The polyenoic, unsaturated PLFA 18:2w6 was used as the indicator of fungal biomass, 16:1w 5c was used as the indicator of AMF, fatty acids containing a 10-methyl group were used as indicators of actinomycetes, and the GP:GN and bacteria:fungi ratios were used as indicators of changes in the relative abundances of these microbial groups [25].

### 2.5. Statistical analyses

Differences between any two treatments in plant biomass (aboveground and root biomasses), soil properties (pH and NO_3_^-^-N, NH_4_^+^-N, and DOC content), or microbial characteristics (total PLFA, MBC, and MBN content and bacterial, fungal, and actinomycete PLFA content) were tested using a one-way blocked analysis of variance with the SPSS statistical software package (SPSS, USA), with each plot representing a block. The data were tested for normality and were logarithmically transformed when required. The homogeneity of the variances was tested by Levene's test. The post hoc separation of means used Duncan’s test. Correlation analysis (Pearson coefficients) tested the relationships between the environmental (plant and soil) parameters and the characteristics of the microbial communities. These analyses were performed using SPSS 20.0.

Structural equation modelling (SEM) was applied to analyse the direct and indirect effects of the environmental variables on the PLFAs, using AMOS 17.0 (Amos Development, Spring House, USA). In a SEM figure each single-headed arrow represents a causal relationship such that the variable at the tail of the arrow is believed to be a direct cause of the variable at the head. An initial SEM is specified based on prior knowledge, and a χ^2^ test is used to determine whether the covariance structures implied by the model adequately fit the actual covariance structures of the data. The model modification indices are a tool for data exploration and hypothesis generation [26]. SEM began with the specification of a conceptual model of hypothetical relationships, based on prior experience and theory. We assumed that N addition would alter soil properties and plant biomass, which in turn would affect the composition and biomass of a microbial community. pH, NH_4_^+^-N, NO_3_^-^-N and DOC content were used as the soil parameters, and plant biomass was divided into aboveground and root biomass. Model fit was evaluated using χ^2^ values, *P*-values (*P* > 0.05), a low value of the Akaike Information Criterion (AIC), and low root square mean errors of approximation (RMSEA), based on the method by Jassey et al. [27].

Before analysis, we tested the detrended correspondence analysis (DCA). The results showed that the variation of axis 1 was 0.32 (< 0.4), so we used the redundancy analysis (RDA). RDA was applied to elucidate the response of microbial community composition to the environmental parameters, which was conducted using CANOCO 4.5 (Microcomputer Power, Inc., Ithaca, USA). Microbial community compositions (PLFAs) were used as the microbial data, and the plant and soil parameters were used as the environmental variables.

## Results

### 3.1. Effect of N addition on plant and soil properties

Aboveground and root biomasses had similar hump-shaped responses to increasing N addition, peaked at 5.6 g N m^-2^ y^-1^ (Table 1). pH was decreased at the level of 22.4 g N m^-2^ y^-1^, significantly. NH_4_^+^-N content tended to decrease as the level of N addition increased and NO_3_^-^-N content increased significantly with increasing N addition. DOC increased with N addition and peaking at 5.6 g N m^-2^ y^-1^.

**Table 1:** Effect of N addition on plant biomass, soil properties and soil microbial characteristics. Number of replicates (n = 5).

N addition rate (g N m^-2^ yr^-1^)	CK	N_2.8_	N_5.6_	N_11.2_	N_22.4_
	0	2.8	5.6	11.2	22.4
Plants (g)					
Aboveground biomass	568±24.0ab	682±83.54ab	773.17±51.5a	734.33±136.3ab	491±34.46b
Root biomass	222±42.78ab	208±78.94ab	274±63.08a	235±55.17ab	191±19.71b
Soils					
pH	8.59±0.04a	8.58±0.04a	8.53±0.04a	8.51±0.12a	8.41±0.08b
NO_3_^-^-N (mg kg^-1^)	1.91±0.26d	2.38±0.61cd	2.96±0.47bc	3.57±1.41b	4.61±0.99a
NH_4_^+^-N (mg kg^-1^)	6.05±0.92a	5.93±2.01a	3.59±0.54b	3.63±1.55b	2.84±0.56b
DOC (mg kg^-1^)	85.00±8.84b	109.53±5.7ab	123.31±8.44a	118.19±6.38a	120.13±10.4a
Microbials					
Total PLFA (nmol g^-1^)	24.839±2.8b	28.50±2.18ab	29.15±2.93ab	32.22±11.56a	32.54±5.14a
MBC (mg kg^-1^)	114±30.43b	98.7±19.71b	106.2±8.32b	100.7±38.78b	222.6±17.18a
MBN (mg kg^-1^)	26.51±3.94c	35.38±3.61c	25.69±5.19c	55.02±5.53b	85.69±6.99a

Note: NO_3_^-^-N, nitrate nitrogen, NH_4_^+^-N, ammonium nitrogen, DOC, dissolved organic carbon, MBC, microbial biomass carbon, MBN, microbial biomass nitrogen.

Data represents the average of five replications ± SE.

Values in a row followed by the different letters are significantly difference among the N treatments (*P* < 0.05).

### 3.2. Effect of N addition on microbial characteristics

**Fig 1:**
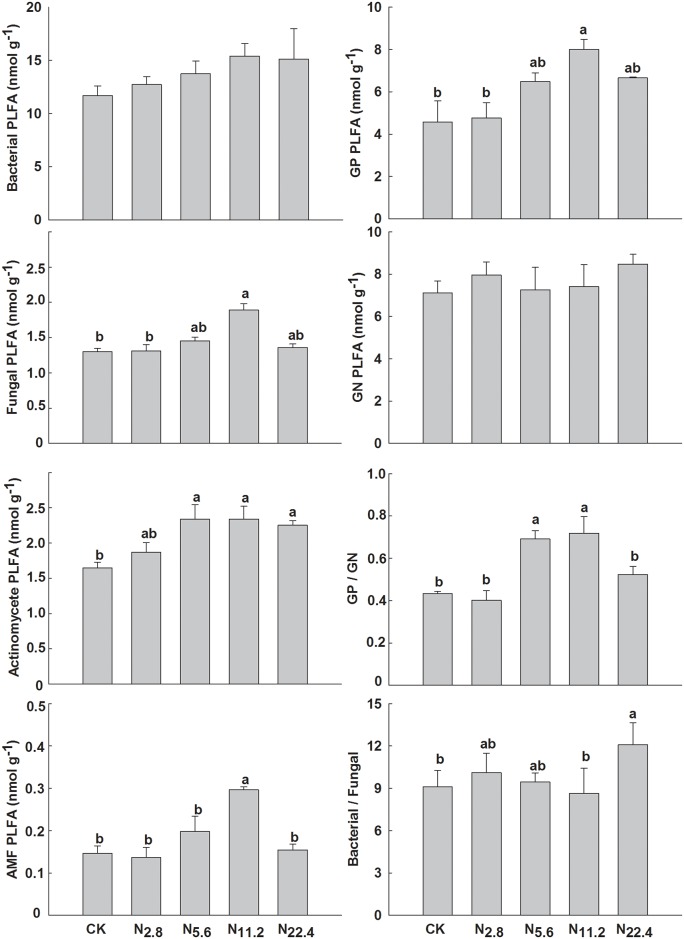
Effect of N addition on phospholipid fatty acid (PLFA) content of different microbial community (mean ± SE). (A) Different letters above bars denote significant difference among treatments (*P* < 0.05). (B) AMF: arbuscular mycorrhizal fungal, GP: gram-positive bacterial, GN: gram-negative bacterial, GP/GN: GP and GN ratios, Bacterial/Fungal: bacterial and fungal ratios.

Lower levels of N addition had no significant influence on the MBC (≤11.2 g N m^-2^ y^-1^) or MBN (≤5.6 g N m^-2^ y^-1^) content, but higher levels increased both MBC (22.4 g N m^-2^ y^-1^) and MBN (≥11.2 g N m^-2^ y^-1^) content (Table 1). Total PLFA content increased with increasing N addition. Nitrogen addition had no significant effect on bacterial or GN abundances (Fig 1). Fungal, AMF, GP, and actinomycete abundances and the GP:GN ratio tended to first increase and then decrease. The bacteria:fungi ratio was significantly higher (*P* < 0.05) at 22.4 g N m^-2^ y^-1^ than other treatments. GN was the most common and stable bacterial group (GP:GN <1).

### 3.3. Effect of N addition on the composition of the microbial communities

**Fig 2:**
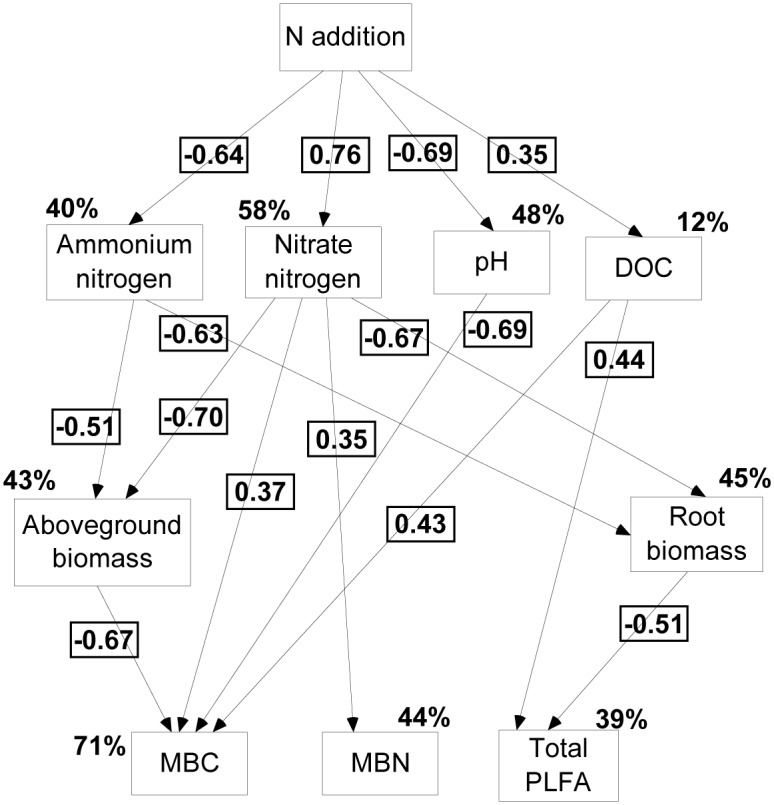
Structural equation model (SEM) of N addition effects on the plant-soil-microbe system. **Examining the multivariate effects on soil microbial total PLFA, MBC, and MBN through hypothetical pathways of soil (nitrate N, ammonium N and DOC content, and pH) and plant (aboveground and root biomass) properties**. The final model fit the data well: χ^2^_15_ = 8.158, P = 0.137, Akaike Information Criteria (AIC) = 130.000, RMSEA = 0.313. Percentages close to endogenous variables indicate the proportion of variation explained by the model (R^2^). Numbers at arrows are standardized path coefficients represent the pathways have significant effects (*P* < 0.05). MBC, microbial biomass carbon, MBN, microbial biomass nitrogen, DOC, dissolved organic carbon.

The SEM described the direct and indirect pathways of variation in total PLFA, MBC, and MBN content (Fig 2, χ^2^_15_ = 8.158, *P* = 0.137). The model suggested that N addition strongly affected the abundances of soil microbial groups and total microbial biomasses (total PLFA content) and microbial activities (MBC and MBN content) by indirect effects on the plant and soil properties. Total PLFA content was directly altered by DOC content and root biomass, based on the significant standardised path coefficients (Fig 2). The DOC content had increased total PLFA content, and root biomass had decreased microbial biomass. Aboveground biomass, NO_3_^-^-N content, DOC content, and pH were directly associated with MBC content, and MBN content was only associated with NO_3_^-^-N content. The correlation analysis indicated that the available-N (NH_4_^+^-N and NO_3_^-^-N) content was significantly correlated only with MBN content. NO_3_^-^-N content was strongly (*P* < 0.01) correlated with MBN content, DOC content was significantly correlated with total PLFA and MBC content, and pH was negatively correlated with MBC and MBN content (Table 2).

**Table 2:** Correlation analysis among the soil resource, plant biomass and microbial biomass.

	NH_4_^+^-N	NO_3_^-^-N	DOC	pH	Aboveground biomass	Root biomass
Total PLFA	-0.22	0.31	0.37*	-0.31	-0.24	-0.37*
MBC	0.02	0.21	0.41*	-0.41*	-0.33	-0.21
MBN	-0.45*	0.60**	0.31	-0.60**	-0.14	-0.11

Note:

* indicated significant correlation (*P* < 0.05).

** indicates highly significant correlation (*P* < 0.01).

**Fig 3:**
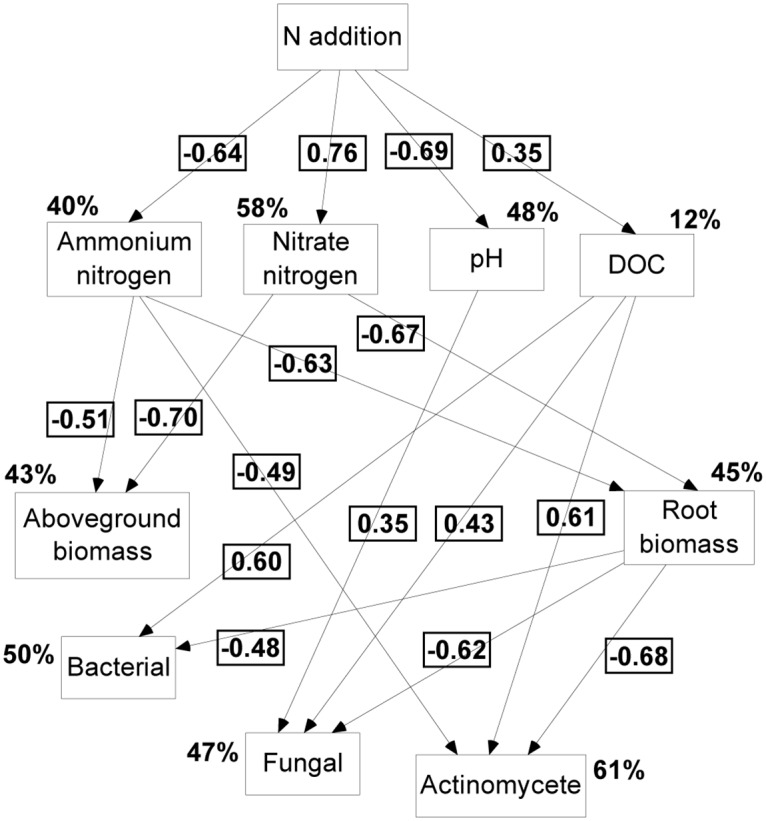
Structural Equation Model (SEM) of N addition effects on bacterial, fungal, and actinomycete. The final model fit the data well: x^2^_15_ = 8.083, P = 0.201, Akaike Information Criteria (AIC) = 130.000, RMSEA = 0.311. Percentages close to endogenous variables indicate the proportion of variation explained by the model (R^2^). Numbers at arrows are standardized path coefficients represent the pathways have significant effects (*P* < 0.05). DOC, dissolved organic carbon.

**Fig 4:**
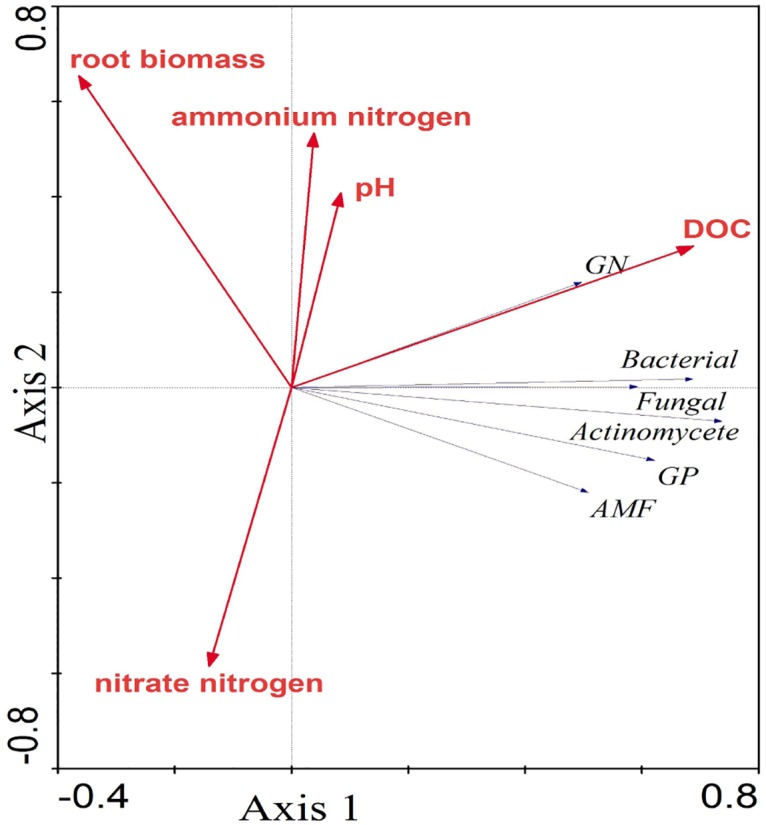
Redundancy Analysis (RDA) of the PLFA data for the 30 soil samples, using 6 PLFAs as species and 6 environmental variables. The arrows indicate the lengths and angles between explanatory and response variables and reflect their correlations. The red lines, blue lines and cycles represent the environmental variables, microbial groups and soil samples, respectively. DOC, dissolved organic carbon, AMF, arbuscular mycorrhizal fungal, GP, gram-positive bacterial, GN, gram-negative bacterial.

The SEM model (Fig 3) indicated that the bacteria, fungi and actinomycetes were all directly altered by DOC content and root biomass. The fungi and actinomycetes were also significantly affected by pH and NH_4_^+^-N content, but the degree of influence was less than by DOC content and root biomass. The RDA plot identified the relationships between the environmental parameters and the microbial community composition (Fig 4). The model (sum of the eigenvalues from the canonical) explained a total of 61.80% of the fitted PLFAs, axis 1 explaining 41.8% and axis 2 explaining 20.0% of this variation, both of which were significant. The first axis indicated that each group was mainly affected by the DOC content, and the second axis indicated that the distribution of the microbes had been strongly influenced by root biomass and NO_3_^-^-N content, but in opposite directions.

## Discussion

### 4.1. Effect of N addition on the characteristics of the plant and soil

Nitrogen addition strongly affected the plant and soil properties (Table 1, Fig 2). Low levels of N addition (<11.2 g N m^-2^ y^-1^) promoted plant growth, peaking at 5.6 g N m^-2^ y^-1^, and high levels of N addition (22.4 g N m^-2^ y^-1^) decreased the aboveground and root biomasses of the *P*. *tabuliformis*, perhaps due to the toxicity to plants of excessive levels of N, which is consistent with the results from Northern China by Wei et al. [18] and Northwest China by Zhong et al. [9]. They found that a threshold rate of 5.6 g N m^-2^ y^-1^ and 18 g N m^-2^ y^-1^ caused the decline of microbial activity and was toxic to plants, respectively. Previous observations also indicated that low levels of N addition, particularly in N-limited ecosystems, generally increased plant biomass, and overloading could suppress plant growth [28]. Nitrogen addition, however, can increase DOC content, perhaps by an increase in the input of plant C content or a decrease in microbial decomposition [18]. Nitrogen addition in our study did not inhibit bacterial, fungal, or actinomycete population abundance (Fig 1), so the higher DOC content with N addition may due to an increase in the distribution of C in the fine roots, fine-root turnover [29], or root exudates [30]. The increase in NO_3_^-^-N content and decrease in NH_4_^+^-N content implied that nitrification gradually increased with N addition, and the NH_4_^+^-N was converted to NO_3_^-^-N. Soil NO_3_^-^-N is usually easily transferred from the upper soil layers to deeper layers if NO_3_^-^-N is not absorbed by the vegetation or incorporated by soil microbes [31]. High levels of N addition significantly decreased soil pH, and soil acidification from N addition has been well documented for grasslands, forests, and other ecosystems [32,33]. DOC content is generally strongly correlated with NH_4_^+^-N consumption [34], and net rates of N mineralisation and nitrification can increase dramatically [35], inducing soil acidification. N addition in our study also significantly decreased soil pH, but not to the level of acidification and dependent on the specific soil property and N-addition level.

### 4.2. Effect of N addition on the characteristics of the microbial communities

Nitrogen addition commonly decreases soil microbial biomass [18,35], but N addition in our study significantly increased total PLFA, MBC, and MBN content at additions of 11.2, 22.4, and 5.6 g N m^-2^ y^-1^, respectively, perhaps associated with the initial N and organic C content of the soil. Soil N content was very low (N levels of CK) in our study area, within the range indicating serious N limitation [29]. N addition thus rapidly increased the available-N content and uniformly increased total PLFA, MBC, and MBN content. DOC content, however, was the main limitation for microbial activities. DOC content in this area is also low, and that is an important food source for microbes. And this consistent with the positive correlation between DOC content and total PLFA and MBC content (Table 2, Fig 2). But in the study area by Hu et al. [35], the soil available-N content was higher (total N content of 7.23g kg^-1^) and organic matter was abundant, so N addition decreased microbial biomass under these conditions, which may also have been associated with soil-saturated N, when microbial growth will no longer be limited to the DOC content or inorganic-N content. pH in our study was negatively correlated with MBC and MBN content (Table 2), suggesting that the decrease in pH from the N addition altered the MBC and MBN content. Available-N (NH_4_^+^-N, NO_3_^-^-N) content was also strongly correlated with MBN content, especially NO_3_^-^-N content. N addition increased the NO_3_^-^-N content and the ability of the microbes to fix N [36]. Total PLFA content was negatively correlated with root biomass (Fig 2), similar to the findings by Marschner and Timonen [22], who suggested that high levels of N addition would inhibit root growth. Root biomass in our study, however, did not differ significantly between CK and the treatment with the highest level of N addition, implying that this negative relationship might be associated with the lower need for plants to invest C in nutrient-absorbing systems, which would induce a shift in C allocation in favour of aboveground biomass at the expense of root biomass [30,37]. Nitrogen addition increased total PLFA content at the microbial-community level mainly by increasing DOC content and decreasing root biomass. The higher MBC and MBN content, however, contributed to the higher DOC content and soil N content and the lower pH.

### 4.3. Effect of N addition on the different components of the soil microbial communities

Nitrogen addition variously affected several components of the microbial communities, including bacterial and fungal biomasses and the GP:GN ratio (Fig 1), which in turn altered the community composition. Bacterial (including GP and GN) biomass did not change significantly over the gradient of N addition, and fungal (including AMF) biomass tended to first increase and then decrease. These results were similar to those in the meta-analysis by Treseder [37] indicating that bacteria were usually not sensitive to the changes in the external environment that could significantly affect the abundance of fungi. Our results also indicated that the bacteria were generally not more sensitive than fungi to N addition and further illustrated that fungi had a competitive advantage over bacteria in decomposing tissues with low content of available C [38]. Fertilisation can increase the proportion of actinomycetes [8] due to a more appropriate soil pH (6.8) for the growth and development of actinomycetes. Our study also found that N addition, especially the higher levels of N addition (>5.6 g N m^-2^ y^-1^) increased the abundance of actinomycetes, but the range of soil pH was not suitable for actinomycetes, implying that the higher levels of N addition alter the pH ranges here did not affect these microbes, thus some other variable associated with the N deposition may have been the driver, for example, root residues [39]. In our study, the bacteria:fungi ratio increased significantly at the highest level of N addition (22.4g N m^-2^ y^-1^) due to the large decrease in AMF abundance. Nitrogen addition is a major driver of decreases in fungal biomass, so the higher ratio also implied a decreased stability of the microbial community. The significantly lower GP:GN ratios were likely due to a lower GP abundance, similar to the results by Siira-Pietikäinen et al. [40], who indicated that nutrient inputs can alter bacterial composition without altering total bacterial biomass. We also found that GN dominated the bacterial communities (GP:GN <1), consistent with a shift from oligotrophic to more copiotrophic soil conditions [41].

Similar to total PLFA content, the SEM model indicated that the bacteria, fungi, and actinomycetes were directly affected by DOC content and root biomass, consistent with the RDA analysis (Fig 4). Aber et al. [42] reported that soil DOC content and shifts in root production could change the distribution of soil microbial species; DOC content became a limiting factor for C sources at high levels of N addition, and the soil microbes depended mainly on the decomposition of roots to meet their requirements for growth and development, further suggesting that DOC and roots play important roles for the supply of C for microbes. We also found that DOC content increased with the abundance of the bacteria, fungi, actinomycetes and were negatively correlated with root biomass. These results do not support those by Li [43] for the effects of N deposition on temperate grassland communities, where roots had no direct effect on bacterial abundance but where DOC had a strong inhibitory effect. Plant litter and roots can reduce the ability to regulate bacterial community composition in grassland ecosystems, but DOC can still accumulate from litter to significantly affect the composition of soil bacterial communities.

*P*. *tabuliformis* are evergreen trees, and their litters have a smaller effect on the input of soil nutrients. Also, mainland soil is nutritionally deficient, so the changes in microbial community composition in our study were likely associated with the original soil or plant properties. Soil microbes in different ecosystems and vegetation conditions will respond differently to N addition. Rousk et al. [44] found that fungal growth increased more than five-fold between high (8.3) and low (4.5) pH, and a pure-culture test also indicated a broad pH range for optimal fungal growth [45]. In our study, soil pH ranged between 8.59 and 8.41, and fungal abundance and pH were significantly positively correlated, also suggesting that the change in soil pH induced by N addition was beneficial to fungal growth. Actinomycete abundance was significantly negatively correlated with NH_4_^+^-N content, perhaps due to the mineralisation of nutrients by the actinomycetes [38,39]. Urea application might thus stimulate nitrification and the accumulation of NO_3_^-^-N and thereby actinomycete abundance.

Multiple factors may affect the composition and structure of microbial communities in natural ecosystems, and the interactions among the factors may modify the response of the communities to individual factors. Nitrogen addition can increase the abundance of rhizospheric bacteria, fungi and actinomycetes, although the different groups responded differently, mainly by increasing the bacteria: fungi ratios due to an increase in the proportion of bacteria, especially GP. Nitrogen addition shifted the composition of the rhizospheric microbial communities by altering soil DOC content and root biomass, consistent with our hypothesis. In this study, the seedling biomass and soil microbial abundance reached maximum at the addition level of 5.6 g N m^-2^ y^-1^. The current rate of N deposition in Northwestern China is still lower (2.5 g N m^-2^ y^-1^) than the overloading levels we tested. The current level of N deposition is therefore beneficial to the growth and development of forest ecosystems. A large increase in N deposition due to anthropogenic activities, however, is predicted for the near future [46]. Studying the effects of N addition at a wide range of levels on the plant-soil-microbe system is therefore important for the further development of terrestrial models for studies of global change, especially for optimizing the management of forest systems.

## Conclusions

In this study, at the microbial-community level, N addition increased the level of total phospholipid fatty acids mainly by increasing the content of DOC and root biomass. Increases in soil microbial biomass carbon and N contributed to the higher DOC and soil N contents and to a lower pH. At the microbial-groups level, bacterial and GN abundances did not change significantly with N addition, but actinomycete abundances increased. Fungal, AMF, and GP abundances and the GP:GN ratio first increased and then decreased with N addition and were highest at the N addition level of 11.2 g N m^-2^ y^-1^. Nitrogen addition increased the bacteria:fungi ratios only at 22.4 g N m^-2^ y^-1^. Nitrogen addition increased the abundances of bacteria, fungi, and actinomycetes mainly by increasing the DOC content and decreasing root biomass, additionally by the declining of pH increased fungal abundances, and by the decreasing ammonium N content to reduced actinomycete abundances. Nitrogen addition shifted the composition of the rhizospheric microbial community mainly by altering the DOC contents and root biomass. The current rate of N deposition (2.5 g N m^-2^ y^-1^) benefits plant growth and is improving the abundances of fungi, arbuscular mycorrhizal fungi, GP, and actinomycetes and the GP:GN ratio.
